# minoTour, real-time monitoring and analysis for nanopore sequencers

**DOI:** 10.1093/bioinformatics/btab780

**Published:** 2021-11-15

**Authors:** Rory Munro, Roberto Santos, Alexander Payne, Teri Forey, Solomon Osei, Nadine Holmes, Matthew Loose

**Affiliations:** School of Life Sciences, Queens Medical Centre, University of Nottingham, Nottingham NG7 2UH, UK; School of Life Sciences, Queens Medical Centre, University of Nottingham, Nottingham NG7 2UH, UK; Microsoft Research, São Paulo, SP 04543-907, Brazil; School of Life Sciences, Queens Medical Centre, University of Nottingham, Nottingham NG7 2UH, UK; School of Life Sciences, Queens Medical Centre, University of Nottingham, Nottingham NG7 2UH, UK; Digital Research Service, Jubilee Campus, University of Nottingham, Nottingham NG8 1BB, UK; DeepSeq, School of Life Sciences, Queens Medical Centre, University of Nottingham, Nottingham NG7 2UH, UK; School of Life Sciences, Queens Medical Centre, University of Nottingham, Nottingham NG7 2UH, UK; DeepSeq, School of Life Sciences, Queens Medical Centre, University of Nottingham, Nottingham NG7 2UH, UK

## Abstract

**Summary:**

minoTour offers a Laboratory Informations Management System (LIMS) system for Oxford Nanopore Technology sequencers, with real-time metrics and analysis available permanently for review. Integration of unique real-time automated analysis can reduce the time required to answer biological questions, including mapping and classification of sequence while a run is in progress. Real-time sequence data require new methods of analysis which do not wait for the completion of a run and minoTour provides a framework to allow users to exploit these features.

**Availability and implementation:**

Source code and documentation are available at https://github.com/LooseLab/minotourcli and https://github.com/LooseLab/minotourapp. Docker images are available from https://hub.docker.com/r/adoni5/, and can be installed using a preconfigured docker-compose script at https://github.com/LooseLab/minotour-docker. An example server is available at http://137.44.59.170.

**Supplementary information:**

Supplementary data are available at *Bioinformatics* online.

## 1 Introduction

High throughput real-time portable sequencing has transformed next generation sequencing from specialized centres to individual laboratories and previously unimaginable locations (Castro-Wallace *et al.*, 2017; Quick *et al.*, 2016). Uniquely, Oxford Nanopore Technologies (ONT) sequencing enables true real-time analysis as sequence data are made available during, as well as after, a run (Loose *et al.*, 2016; Payne *et al.*, 2021). Rapid analysis of data provides advantages where time to answer is important, such as pathogen genomics and clinical diagnosis of disease (Goes *et al.*, 2019; Martignano *et al.*, 2021; Quick *et al.*, 2016; Sone *et al.*, 2019).

During a sequencing run, sequencing is controlled and tracked via ONT’s MinKNOW software. MinKNOW can be used to remotely observe and monitor the progress of sequencing runs, view reports on metrics and recent historical run data. Numerous tools provide analysis of nanopore FASTQ data either during or after a run (Bruno *et al.*, 2021; De Coster *et al.*, 2018). ONT also provides a cloud based service, epi2me (ONT, 2021), which enables various automated downstream analyses. However, as we show below, minoTour uniquely can capture both real-time sequence data and run metrics, can provide analysis and also acts as a comprehensive run archive.

minoTour is open source and extensible, written using the Django framework, providing real-time insights into sequencer performance and sequence analysis. Visualized metrics allow users to see flow cell performance in real time. Built-in pipelines for alignment and metagenomics allow users to see experimental results in real time. Additional pipelines can easily be incorporated. For example, we can include a customized version of the ARTIC pipeline for SARS-CoV2 analysis described in more detail elsewhere (Munro *et al.*, 2021). minoTour also provides built-in adaptive sequencing (ReadFish only) (Payne *et al.*, 2021) support in visualizations enabling users to monitor targeted sequencing in real time.

## 2 Materials and methods

**Fig. 1.**
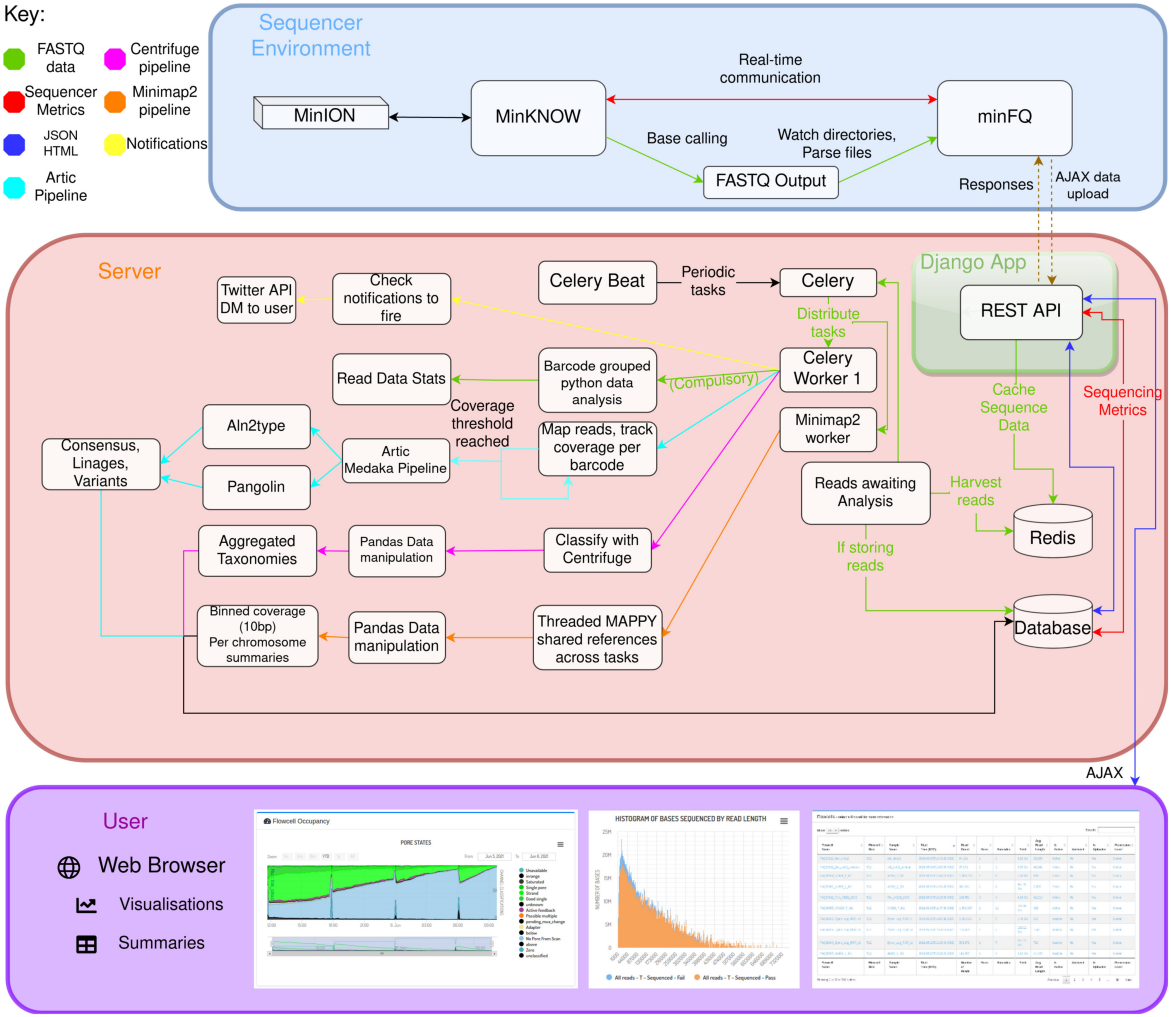
Implementation of minoTour showing the flow of base-called FASTQ data and sequencer metrics from the sequencer to the server, through various optional pipelines and finally to visualization for the user

Figure  1 illustrates the path data takes from the sequencer to the user. A single python tool, minFQ (https://github.com/LooseLab/minotourcli), collects data from two sources. Firstly, run metrics are collected from MinKNOW via an ONT provided application programming interface (API). Secondly, base-called data are read either from user specified locations or found via the ONT API. These data are collected independently of each other, with only one needed to create the flowcell entry. They are sent to the minoTour server, which saves the sequencer metrics into the database, and stores the sequence data in Redis, a cache database. Celery performs asynchronous analysis of read data and can apply custom pipelines to perform analyses, ranging from base-called data summarizing to sequence alignment, metagenomics or custom workflows such as ARTIC (Munro *et al.*, 2021; Tyson *et al.*, 2020). Read data are split into individual flow cells, which can be selected from a table to show all data held for individual runs using that flow cell (Supplementary  Fig.  S1). These data can optionally be shared with other minoTour users if desired (Supplementary  Fig.  S2). minoTour can also be configured to use twitter/email APIs and notify users about flow cell events such as disk shortages or reaching prespecified coverage thresholds for a reference genome (Supplementary  Figs S3  and  S4). Reverse communication with the MinKNOW API allows users to send messages back into the MinKNOW logs for a permanent record such as notes on library reloading or comments on flow cell performance. 

## 3 Results and discussion

One of minoTour’s strengths is its ability to automatically provide a historic record of runs (Supplementary  Fig.  S1), and provide real-time key insights into ongoing runs (Supplementary  Figs  S5  and  S6). minoTour also provides a simple overview of all ongoing connected sequencers, quickly showing a user if any are under-performing, such as falling short on yield or speed, who can then respond accordingly (Supplementary  Fig.  S7). minoTour can also remotely stop a run if required via the website. Using minoTour’s minimap2 alignment pipeline for monitoring targeted or adaptive sequencing with ReadFish one can rapidly tell if coverage is accruing over specific targeted regions with sequenced and rejected reads able to be visualized separately (Supplementary  Fig.  S8).

minoTour provides a simple metagenomics pipeline, using centrifuge (Kim *et al.*, 2016) to visualize sample make-up, a breakdown of broad composition as well as more detailed investigation of select prechosen species by aligning reads classified in that taxa against one or more references (Supplementary  Fig.  S9). More complex pipelines such as the ARTIC pipeline can be incorporated (Supplementary  Fig.  S10). We routinely use minoTour in the laboratory to monitor sequencing and use the available analyses to provide insights into sequencing experiments. The use of the Django framework enables other users to extend and develop minoTour at will. minFQ, once installed and activated, remains in the background uploading data to the minoTour server, automatically detecting new runs and sequence data (Supplementary  Fig.  S11).

Our implementation of minoTour with docker alongside detailed server installations for development leave the user with a variety of installation options. minoTour has been run on large centralized servers, or locally on laptops, enabling monitoring of sequencing runs for collaborative groups, sequencing facilities and individual users. At this time minoTour supports all platforms running flongle or minION flowcells. minoTour currently supports promethION for live data monitoring. Base-called data can be uploaded provided a suitably powerful server is running the app. Analysis pipelines will run, but will most likely fall behind the volumes of data that can be generated by a promethION.

## Supplementary Material

btab780_supplementary_data
