# Supplementary material for: minoTour, real-time monitoring and analysis for nanopore sequencers
Source: Bioinformatics. 2021 Nov 15;38(4):1133–5. doi: 10.1093/bioinformatics/btab780 (PMC8796371; doi:10.1093/bioinformatics/btab780)
Supplement: btab780_supplementary_data [file btab780_supplementary_data.pdf]

Supplementary figures.

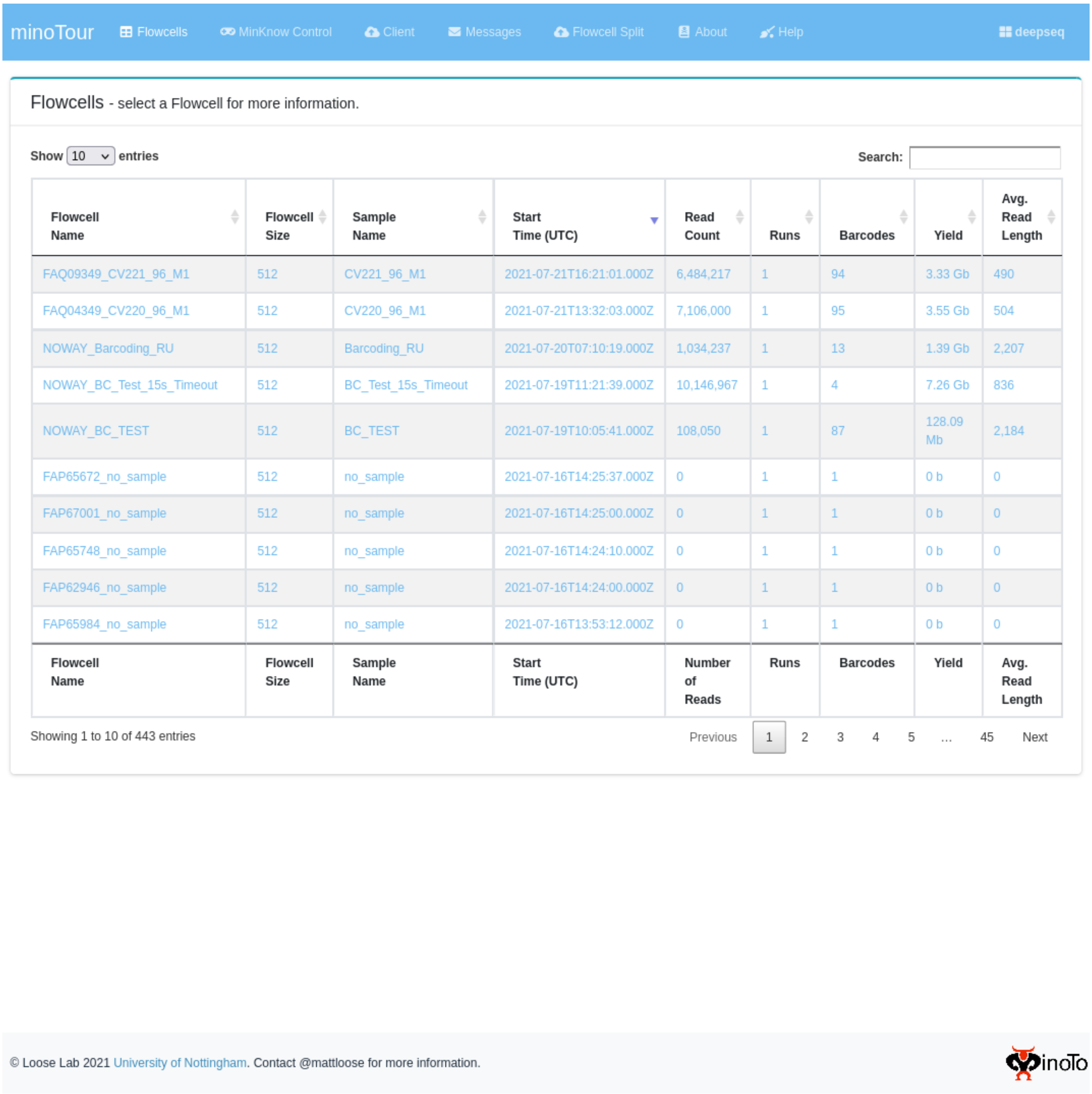

Flowcell Name	Flowcell Size	Sample Name	Start Time (UTC)	Read Count	Runs	Barcodes	Yield	Avg. Read Length
FAQ09349_CV221_96_M1	512	CV221_96_M1	2021-07-21T16:21:01.000Z	6,484,217	1	94	3.33 Gb	490
FAQ04349_CV220_96_M1	512	CV220_96_M1	2021-07-21T13:32:03.000Z	7,106,000	1	95	3.55 Gb	504
NOWAY_Barcoding_RU	512	Barcoding_RU	2021-07-20T07:10:19.000Z	1,034,237	1	13	1.39 Gb	2,207
NOWAY_BC_Test_15s_Timeout	512	BC_Test_15s_Timeout	2021-07-19T11:21:39.000Z	10,146,967	1	4	7.26 Gb	836
NOWAY_BC_TEST	512	BC_TEST	2021-07-19T10:05:41.000Z	108,050	1	87	128.09 Mb	2,184
FAP65672_no_sample	512	no_sample	2021-07-16T14:25:37.000Z	0	1	1	0 b	0
FAP67001_no_sample	512	no_sample	2021-07-16T14:25:00.000Z	0	1	1	0 b	0
FAP65748_no_sample	512	no_sample	2021-07-16T14:24:10.000Z	0	1	1	0 b	0
FAP62946_no_sample	512	no_sample	2021-07-16T14:24:00.000Z	0	1	1	0 b	0
FAP65984_no_sample	512	no_sample	2021-07-16T13:53:12.000Z	0	1	1	0 b	0
Flowcell Name	Flowcell Size	Sample Name	Start Time (UTC)	Number of Reads	Runs	Barcodes	Yield	Avg. Read Length

Supplementary figure 1 - Flow cell table. The landing page for an authenticated user, displaying quick metrics about flow cells.The table is sortable and searchable. Upon clicking a row, the user is redirected to a tabbed page displaying all data held about the flow cell.

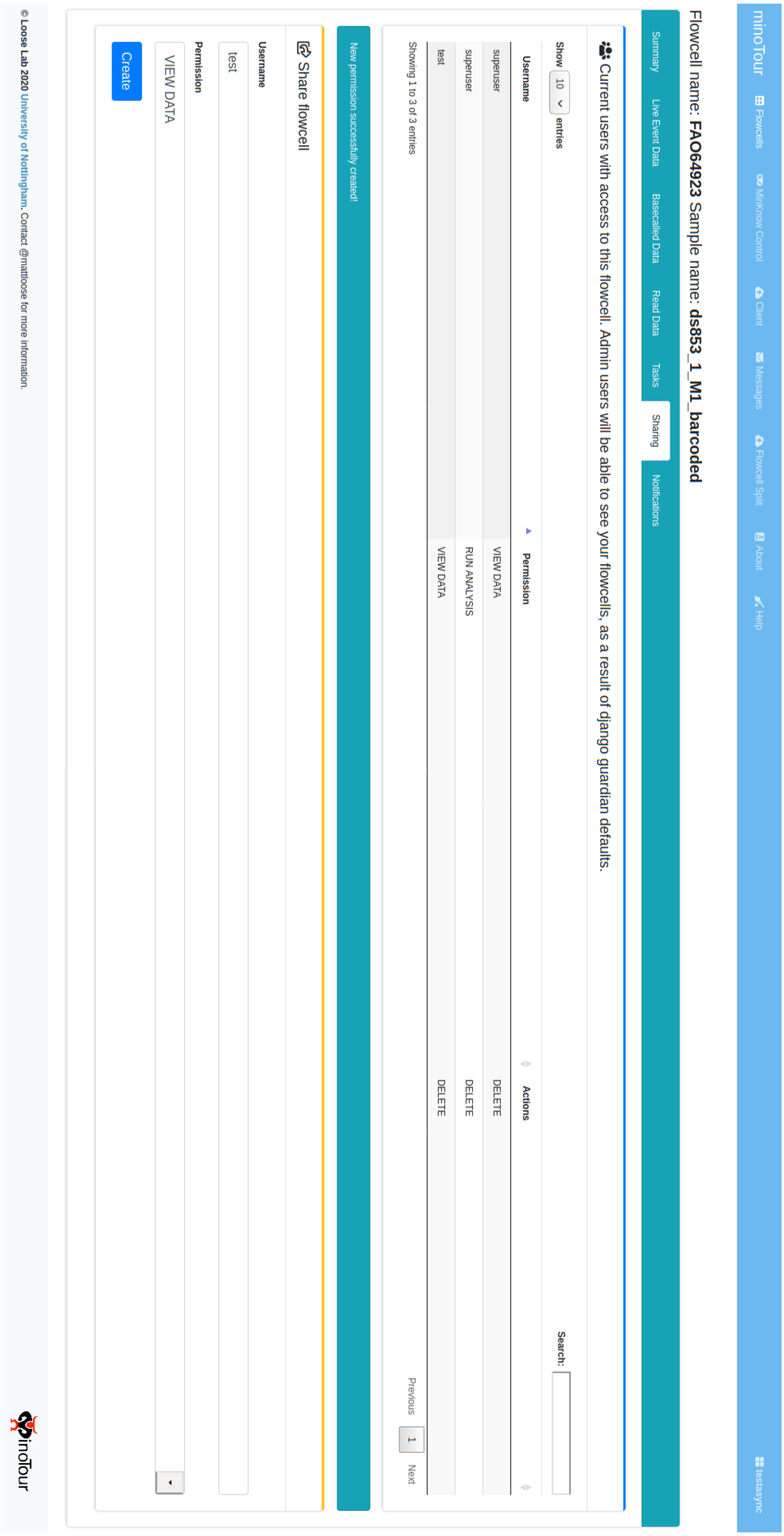

Supplementary figure 2 - Sharing tab. Uploaded flow cells can be shared with other users, with a permission level of either VIEW DATA, or RUN ANALYSES. The sharing permission can later be deleted. Admin users can see all flow cells that have been shared, due to an unalterable default in the package used to manage sharing permissions.

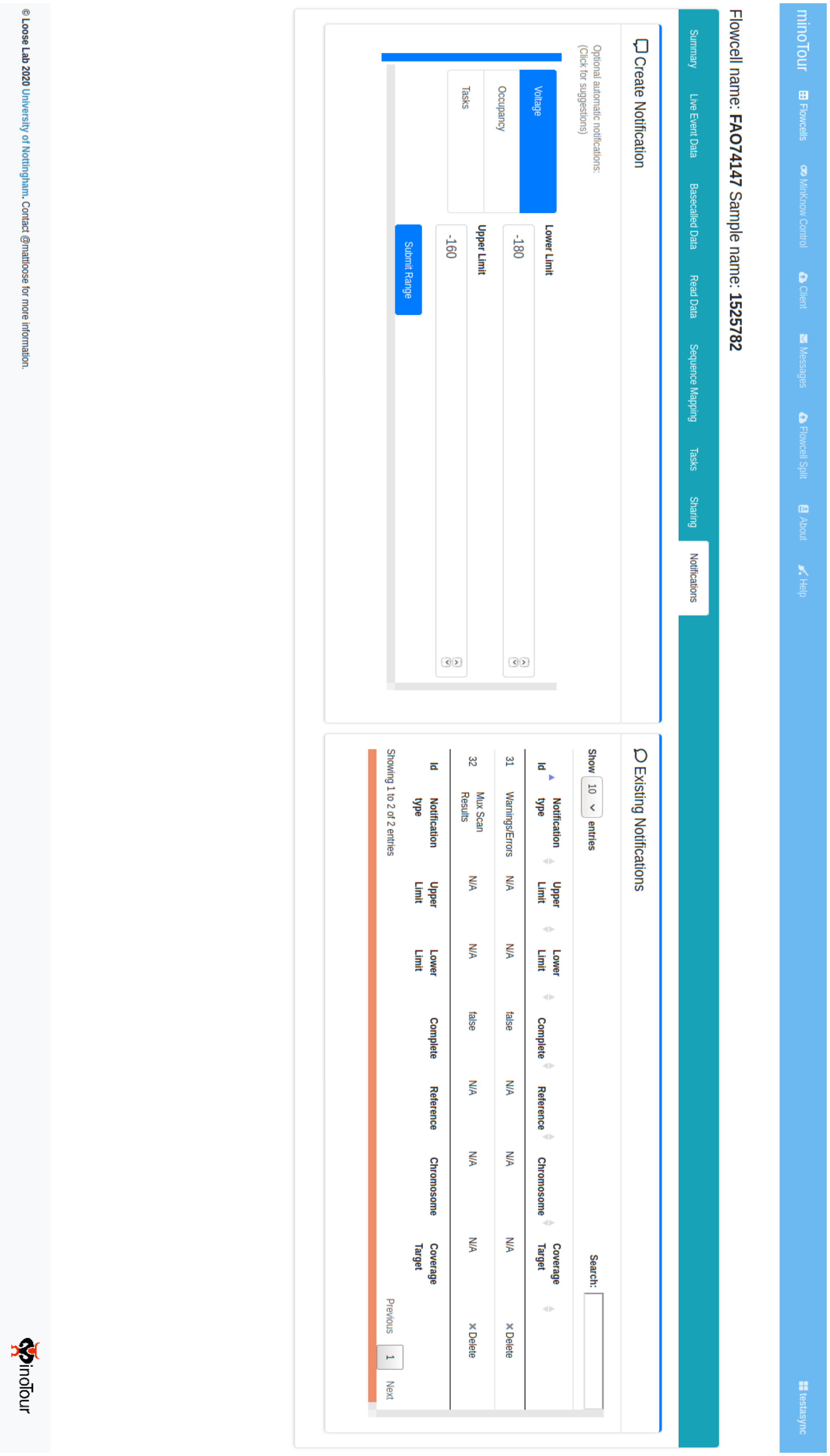

Supplementary figure 3 - Notifications tab. Users can enable notifications that are triggered when certain conditions are met. For example when MinKNOW performs a mux scan, the results of this mux scan are sent to the user via Twitter, if the user has provided their twitter details.

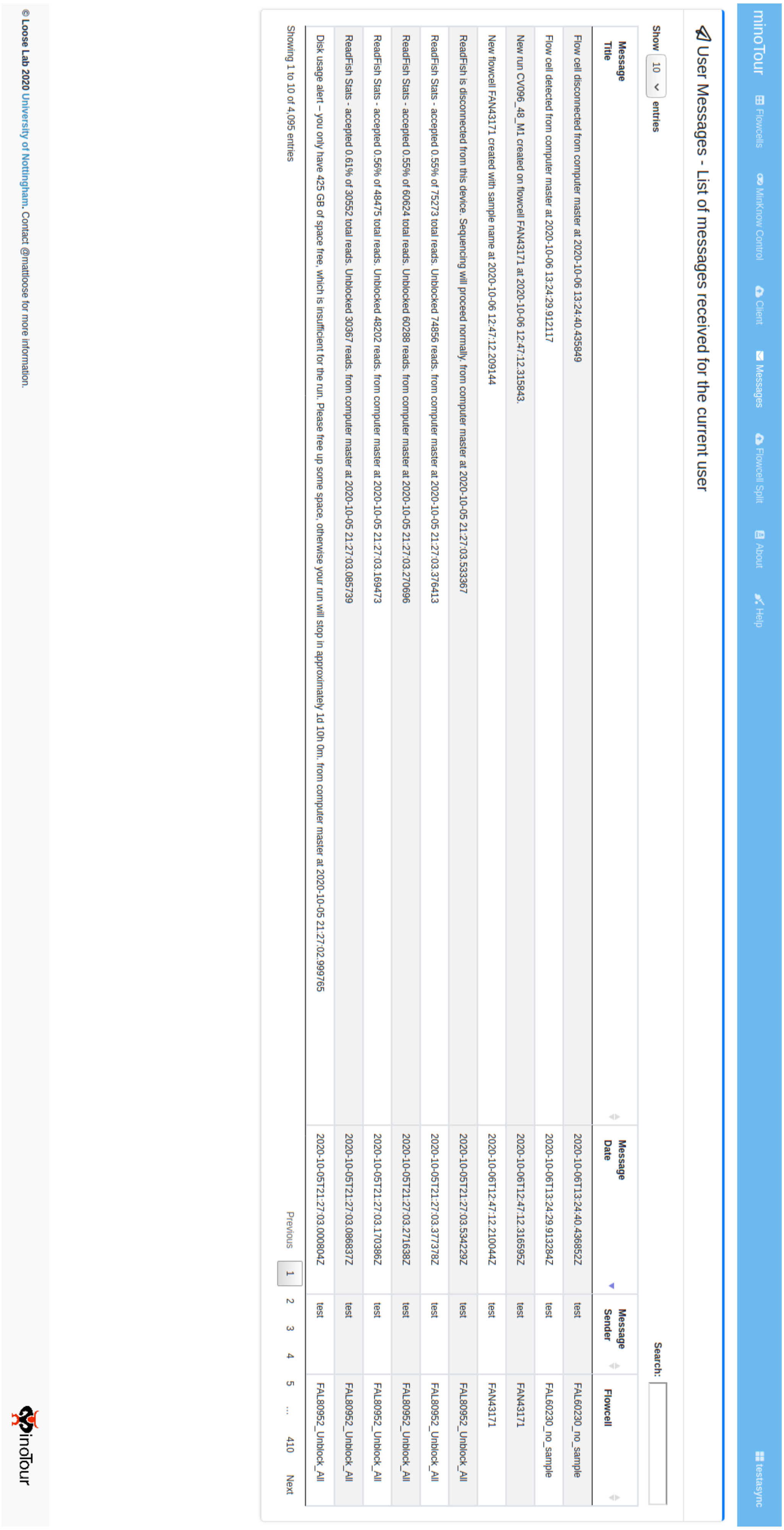

Supplementary figure 4 - Messages page. When notification conditions are met, or when a new flow cell or sequencing run is created in minoTour, a message is created inside the system. This message is then tweeted to the user, alerting them to certain events in the system.

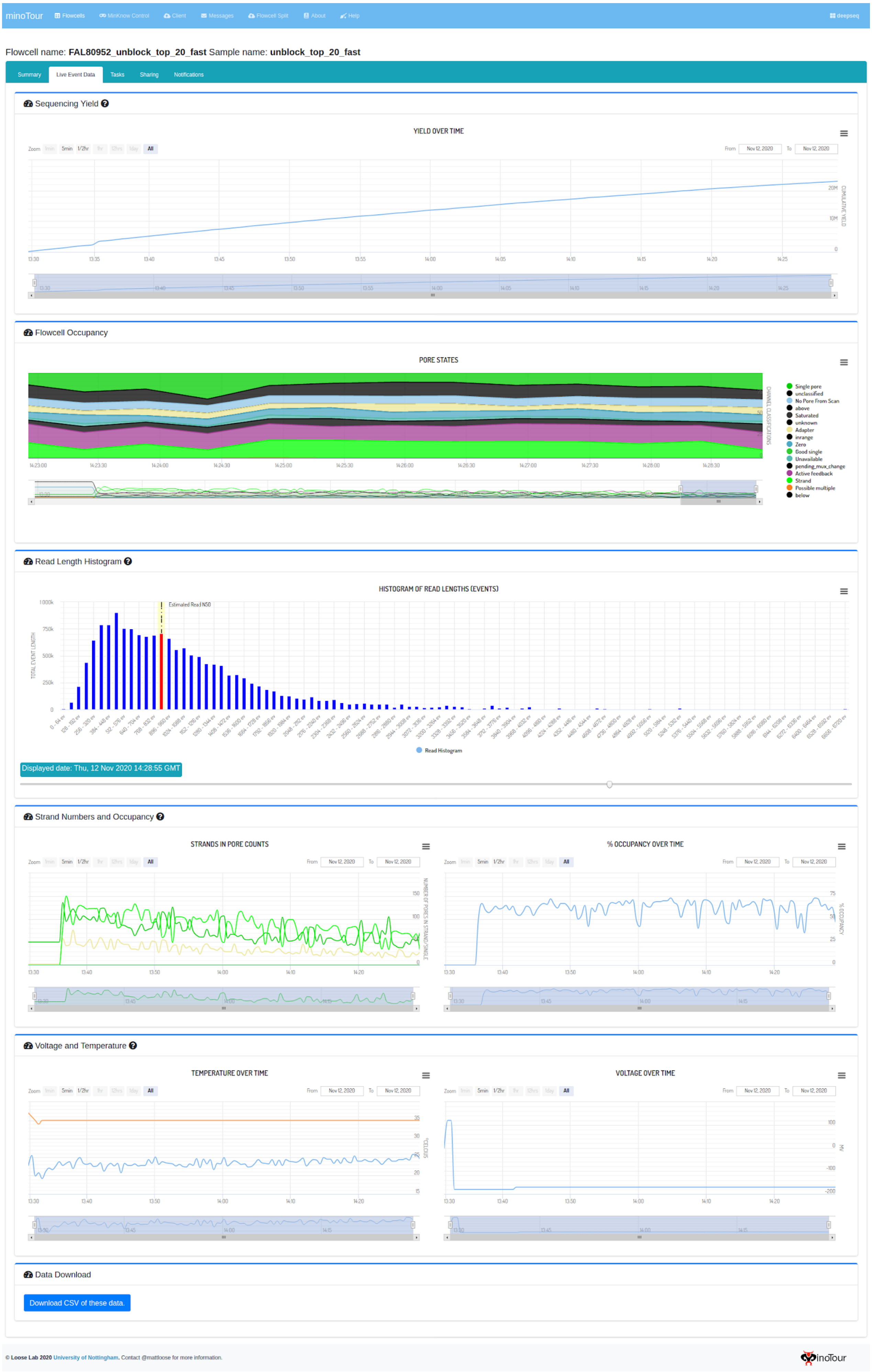

Supplementary figure 5 - Live sequencing metrics tab. This page visualises several sequencing metrics historically, allowing a perfect record of sequencing runs to be kept.

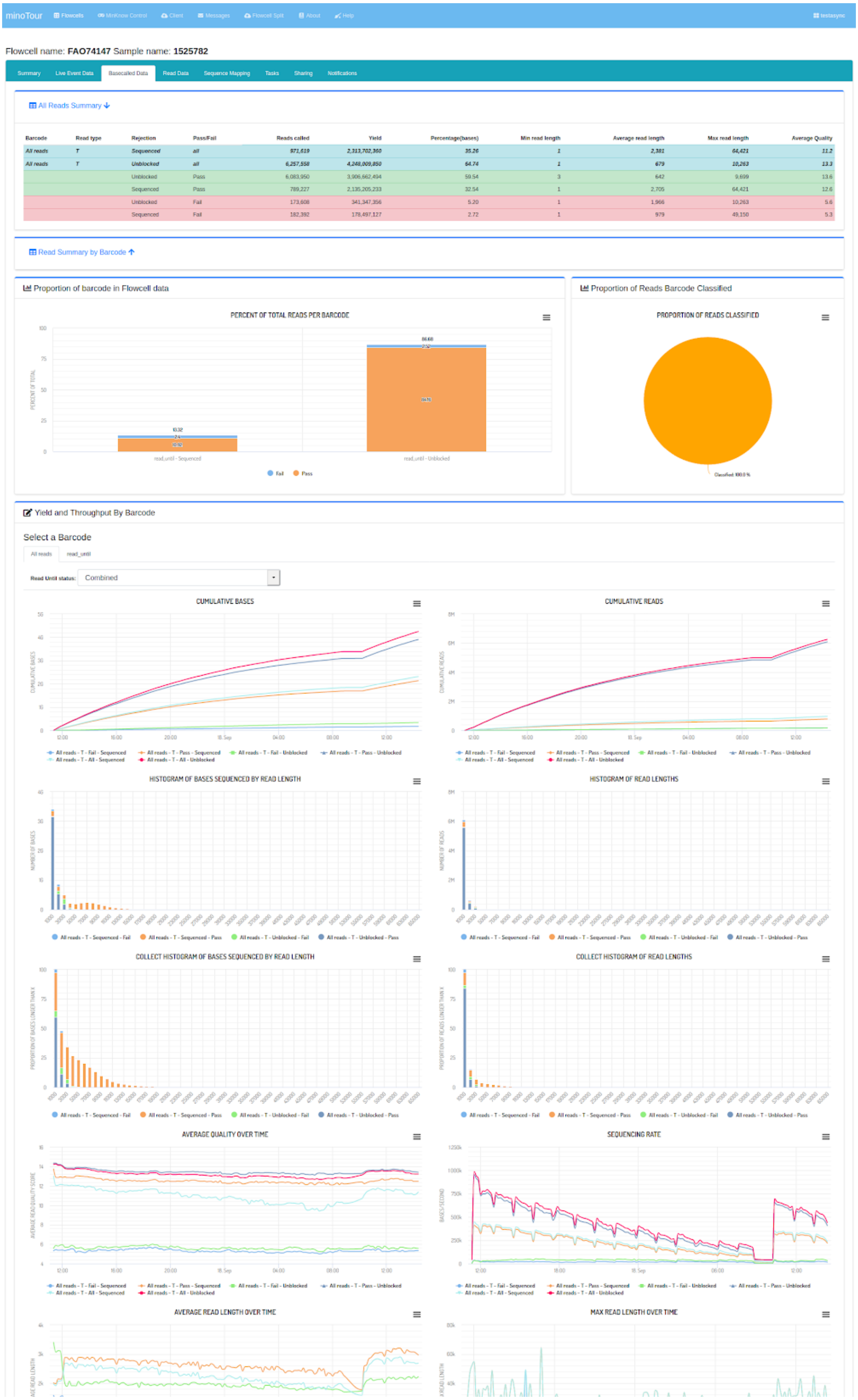

Supplementary figure 6 - Base-called data tab. A summary of base called data produced by a sequencing run. This data can be uploaded separately or not at all. Omitted are a tabular summary of data for each barcode and heatmaps of the flow cell showing yield and read count per pore.

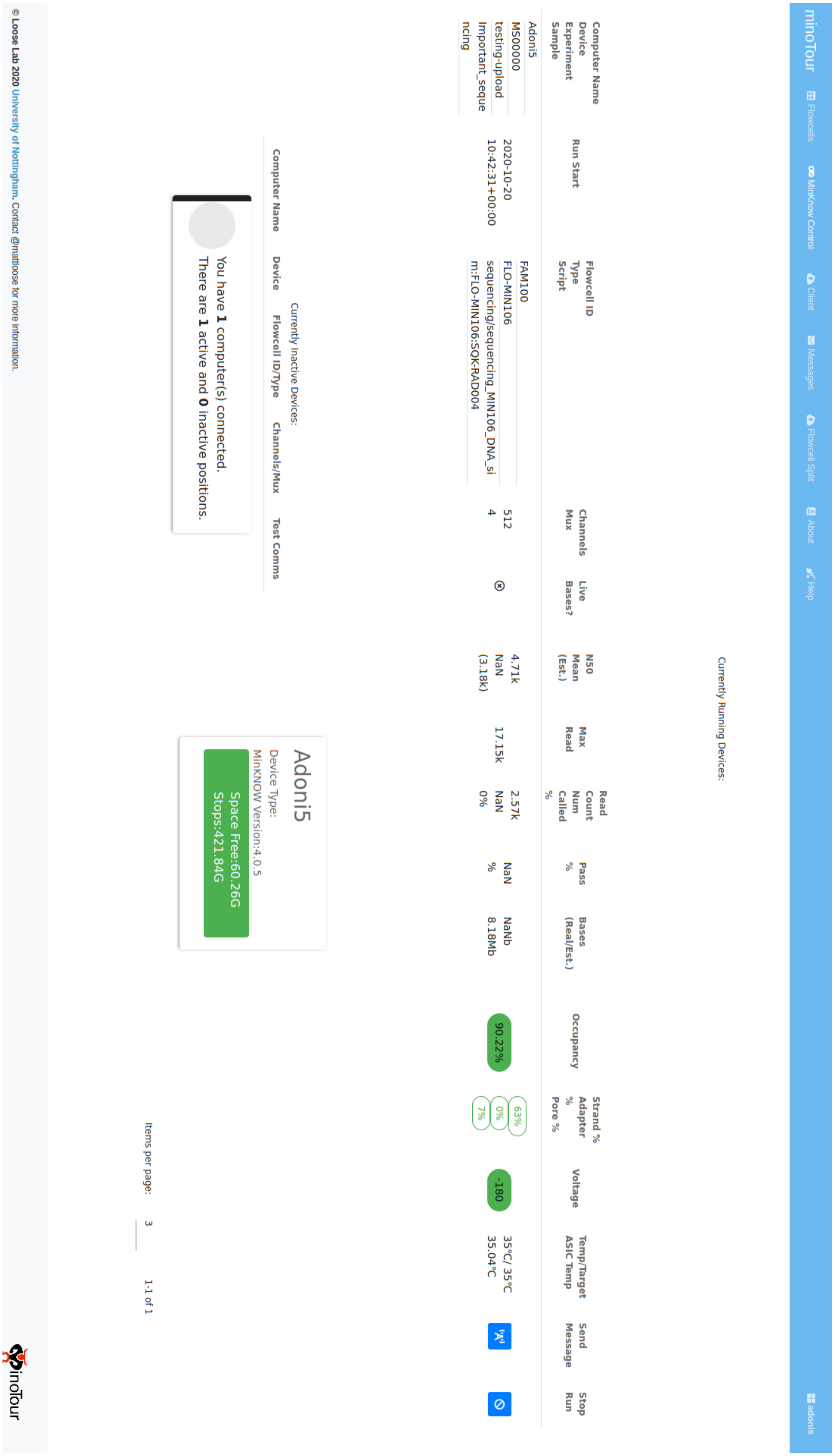

Supplementary figure 7 - MinKNOW control. MinoTour offers comprehensive LIMS capabilities. Demonstrated here is the ability to monitor the live performance of all connected devices, easily flagging any issues, send custom messages to the MinKNOW instance that the device is connected to and remotely stop a run.

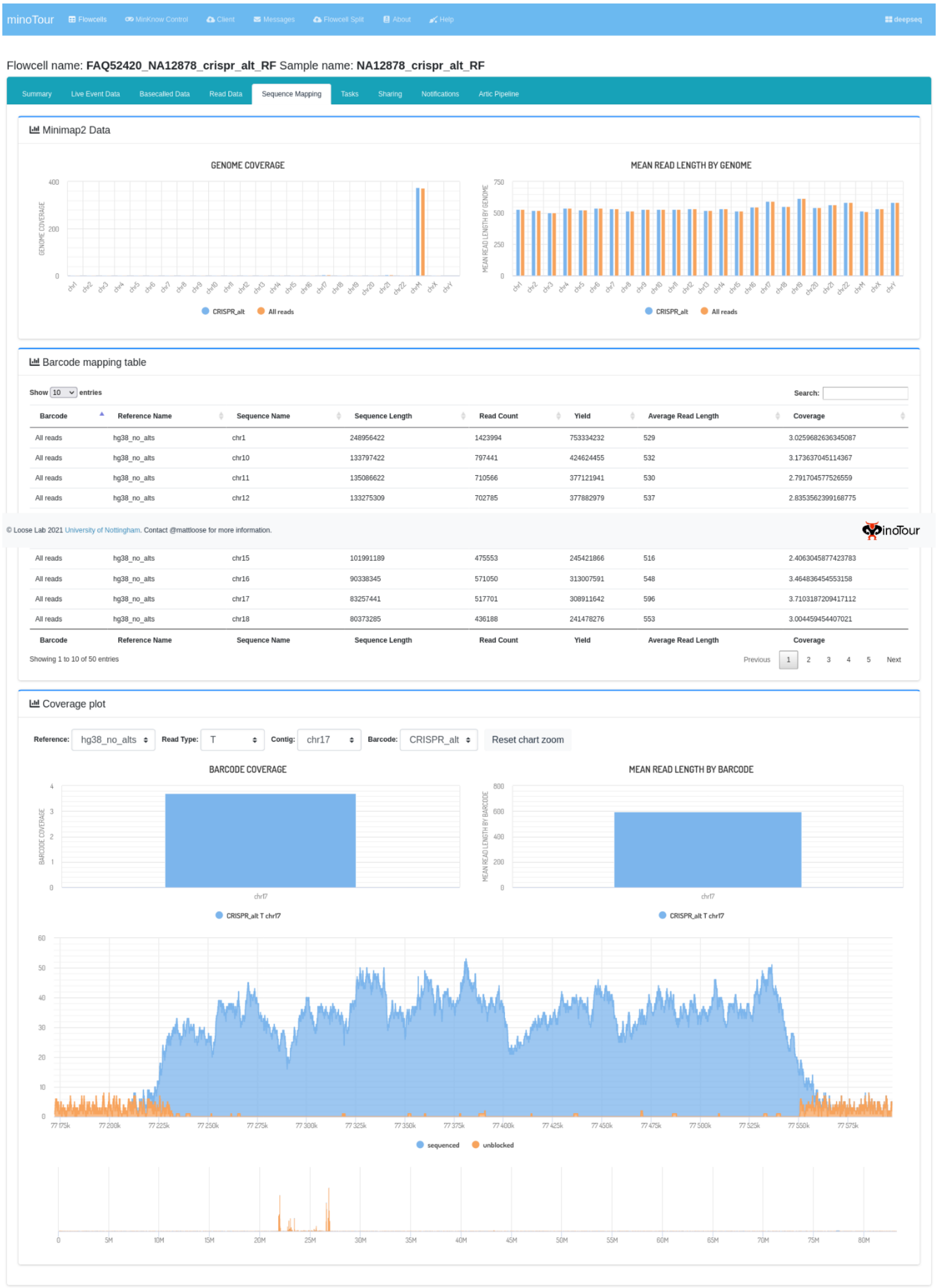

Supplementary figure 8 - Alignment tab. The alignment analysis in minoTour offers a quick insight into a sequencing run in real time, aligning to user specified references. The alignments are broken down by barcode and contig (if multiple). This is not intended as a final result, it is merely to flag any areas that would be interesting for further study. In adaptive sequencing, unblocked and sequenced reads are separately visualised.

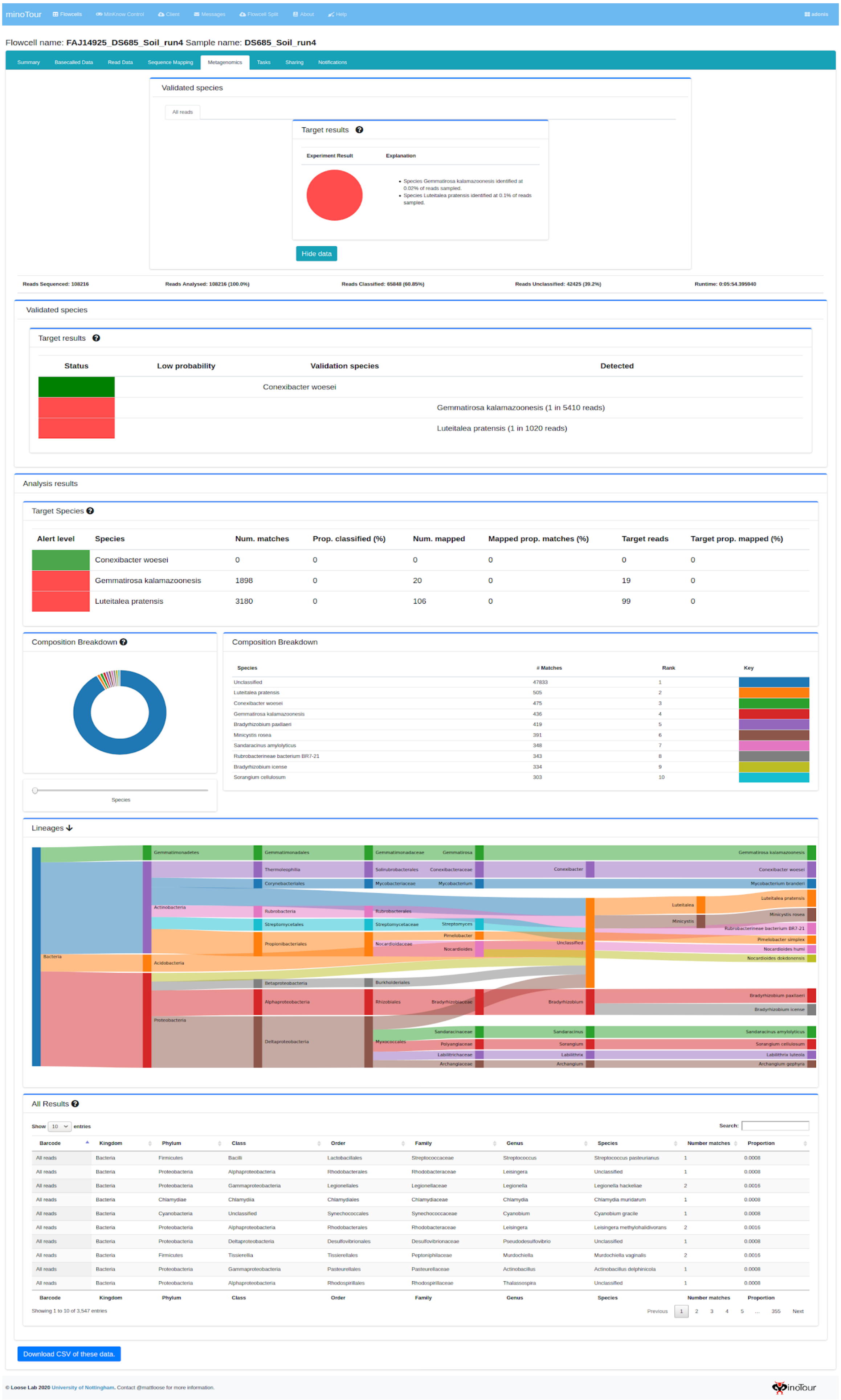

Supplementary figure 9 - Metagenomics tab. The metagenomics analysis is a quick insight into the broader composition of a dataset. Visualisations include a Sankey Diagram denoting the lineages of the 30 most common species by read number, a composition breakdown at each taxonomic level and full results table that is searchable and sortable.

minoTour | Flowcells | MinKnow Control | Client | Messages | Flowcell Split | About | Help | adonis

Flowcell name: **FAQ01023_CV197_25_M1** Sample name: **CV197_25_M1**

Summary | Basecalled Data | Read Data | Tasks | Sharing | Notifications | Artic Pipeline

Artic Coverage Charts

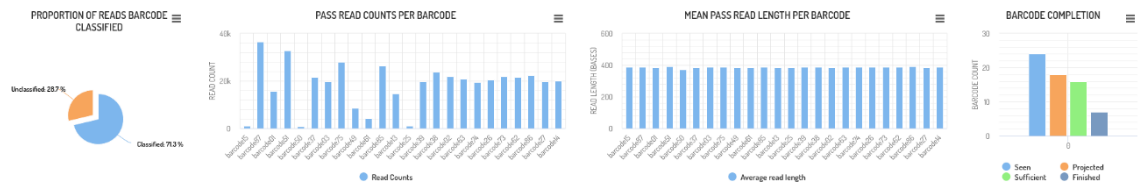

Artic Phylogeny Reports

A SNIPIT plot (see https://github.com/aineniamh/snipit) for consensus sequences for samples completed so far.
Sequences will be added or updated as consensus sequences are generated over time. When a new sequence is generated, the most complete will be used in this plot.
Only sequences with less than 50% N calls are included in this analysis.

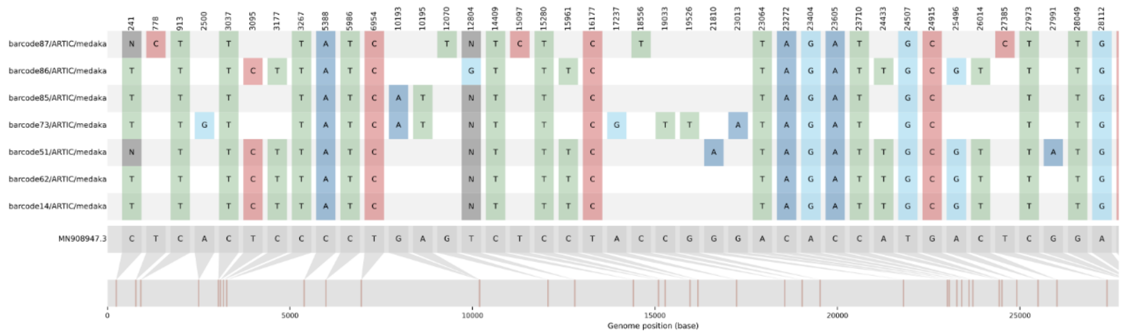

A simple phylogenetic tree for sequences. Trees are generated using iqtree after alignment with mafft. The reference sequence provided for the artic pipeline is used as an outgroup. Trees are visualised using figtree.js.
Only sequences with less than 50% N calls are included in this analysis. Use the slider to adjust the height of the tree image.

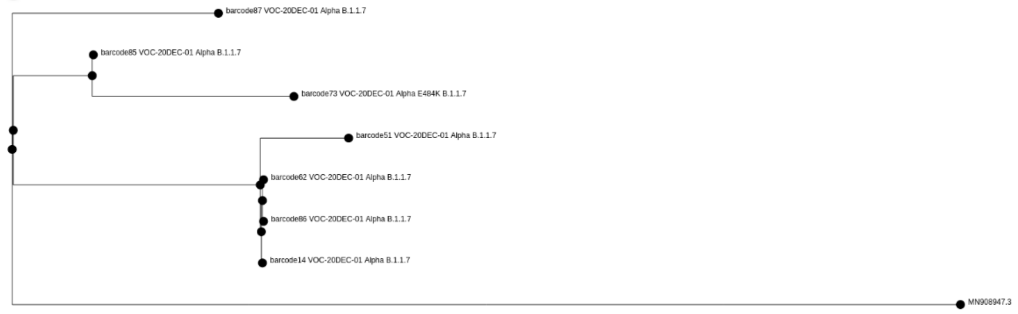

Pipeline firing conditions

20 X Coverage at 90 % of amplicons | Submit choices

Show 10 entries | Search:

% of amplicons	X coverage	Delete
90	20	✕ Delete
**% of amplicons**	**X coverage**	**Delete**

Showing 1 to 1 of 1 entries | Previous | 1 | Next

Summary Table (Pass reads only)

Copy | CSV | PDF | Show 10 rows | Search: barcode

Barcode	Sequence Name	Sequence Length	Read Count	Yield	Average Read Length	Coverage	# Success Amplicons	# Partial Amplicons	# Failed Amplicons	Mean of amplicons	Var of amplicons	Amplicon Std. Dev	Sufficient Coverage	Projected to Finish	Lineage	VoC Found
barcode15	MN908947.3	29,903	1,137	440.68 kb	388	14.45	16	4	79	13.4	1102.03	33.2	false	false	Currently unknown	Not Tested
barcode87	MN908947.3	29,903	36,440	14.12 Mb	388	463.22	99	0	0	445.87	29669.97	172.25	true	true	B.1.1.7	VOC-20DEC-01 (confirmed)
barcode01	MN908947.3	29,903	15,766	6.09 Mb	386	199.52	71	9	19	188.82	60892.39	246.76	false	false	Currently unknown	Not Tested
barcode51	MN908947.3	29,903	32,764	12.81 Mb	391	420.15	99	0	0	404.61	26610.79	163.13	true	true	B.1.1.7	VOC-20DEC-01 (confirmed)
barcode50	MN908947.3	29,903	705	262.70 kb	373	8.61	10	9	80	8.46	887.69	29.79	false	false	Currently unknown	Not Tested
barcode37	MN908947.3	29,903	21,563	8.31 Mb	386	272.39	89	6	4	261.48	46975.64	216.74	false	true	Currently unknown	Not Tested
barcode03	MN908947.3	29,903	19,712	7.63 Mb	387	249.89	92	7	0	243.16	24450.24	156.37	true	true	Currently unknown	Not Tested
barcode75	MN908947.3	29,903	28,058	10.87 Mb	388	356.25	95	4	0	342.77	39780.85	199.45	true	true	Currently unknown	Not Tested
barcode49	MN908947.3	29,903	8,602	3.31 Mb	384	108.39	56	13	30	108.21	22371.43	149.57	false	false	Currently unknown	Not Tested

Barcode	Sequence Name	Sequence Length	Read Count	Yield	Average Read Length	Coverage	# Success Amplicons	# Partial Amplicons	# Failed Amplicons	Mean of amplicons	Var of amplicons	Amplicon Std. Dev	Sufficient Coverage	Projected to Finish	Lineage	VoC Found
barcode61	MN908947.3	29,903	4,319	1.67 Mb	386	54.75	32	6	61	49.38	8000.96	89.45	false	false	Currently unknown	Not Tested

Showing 1 to 10 of 24 entries (filtered from 25 total entries)

Previous 1 2 3 Next

Download all completed | Run all Incomplete | Re-Run All | Export report

Lineage Information for barcode87/ARTIC/medaka - B.1.1.7

Links to information on lineage B.1.1.7

PANGO Info | Outbreak Info

Variant of Concern Report for barcode87/ARTIC/medaka

If a genome is analysed, this will provide a report for the variant or variants found based on current PHE Variant Definitions. This analysis will report all possible VoCs and caution should be taken in interpretation of low coverage/quality genomes.

Reported VuI/VoCs:

sample_id	phe-label	unique-id	status	mutation-ref-calls	mutation-mixed-calls	mutation-calls	indel-ref-calls	indel-calls	no-calls	no-calls-deletion
barcode87/ARTIC/medaka	VOC-20DEC-01	denture-daughter	confirmed	0	0	13	0	2	0	0

Observed mutations:

type	snp	snp	snp	snp	snp	snp	snp	del	snp	snp	snp	snp	snp	snp	del
reference-base	T	C	C	C	C	C	T	GTCTGGTTTT	G	C	T	C	T	C	ATACATG
variant-base	C	T	T	T	A	T	C	G	T	T	C	T	C	T	A
var-length	1	1	1	1	1	1	1	10	1	1	1	1	1	1	7
one-based-reference-position	778	913	3037	3267	5388	5986	6954	11287	12070	14408	15096	15279	16176	18555	21764
iupac-variant-bases	C	T	T	T	A	T	C	G	T	T	C	T	C	T	A

type	del	snp	snp	snp	snp	snp	snp	snp	snp	snp	snp	snp	del	mnp	mnp-snp	mnp-snp
reference-base	TTTA	A	C	A	C	C	T	G	T	C	G	A	TA	GAT	A	T
variant-base	T	T	A	G	A	T	G	C	C	T	T	G	T	CTA	T	A
var-length	4	1	1	1	1	1	1	1	1	1	1	1	2	3	1	1
one-based-reference-position	21990	23063	23271	23403	23604	23709	24506	24914	27384	27972	28048	28111	28270	28280	28281	28282
iupac-variant-bases	T	T	A	G	A	T	G	C	C	T	T	G	T	CTA	T	A

Sample_ID: barcode87/ARTIC/medaka
PANGO:B.1.1.7
nextstrain:N501Y.V1

Variant Status: confirmed

PHE-Label: VOC-20DEC-01
WHO Label: Alpha
Alternate Names: VOC202012/01, UK variant, Kent variant, VOC1,

Description:
This variant became widespread in the UK in the Winter of 2021 and is characterised by increased transmissibility.
Information Sources Source_1 Source_2

Variant Calls Detected:

Position	gene	protein	ref	variant	sample call	type	status
3267	ORF1ab	nsp3	C	T	T	SNP	detect
5388	ORF1ab	nsp3	C	A	A	SNP	detect
6954	ORF1ab	nsp3	T	C	C	SNP	detect
21764	S	surface glycoprotein	ATACATG	A	A	deletion	detect
21990	S	surface glycoprotein	TTTA	T	T	deletion	detect
23063	S	surface glycoprotein	A	T	T	SNP	detect
23271	S	surface glycoprotein	C	A	A	SNP	detect
23604	S	surface glycoprotein	C	A	A	SNP	detect
23709	S	surface glycoprotein	C	T	T	SNP	detect
24506	S	surface glycoprotein	T	G	G	SNP	detect
24914	S	surface glycoprotein	G	C	C	SNP	detect
27972	ORF8	ORF8 protein	C	T	T	SNP	detect
28048	ORF8	ORF8 protein	G	T	T	SNP	detect
28111	ORF8	ORF8 protein	A	G	G	SNP	detect
28280	N	nucleocapsid phosphoprotein	GAT	CTA	CTA	MNP	detect

One based position - notes on sites detected in this sample.

The observed calls were:

- mutation_ref_calls: 0
- indel_ref_calls: 0
- mutation_calls: 13
- mutation_mixed_calls: 0
- indel_calls: 2
- no_calls: 0
- no_call_deletion: 0

Classification rules:

confirmed	probable	low_qc
mutations_required: 13	mutations_required: 5	mutations_required: 0
indels_required: 0	indels_required: 0	indels_required: 0
allowed_wildtype: 0	allowed_wildtype: 0	allowed_wildtype: 0

Acknowledgements:
*Curators:*Natalie Groves Ulf Schaefer Nick Loman

The complete list of variants of concern scanned included:
VUI-21FEB-04 VOC-20DEC-02 VUI-21FEB-01 VUI-21MAR-01 VUI-21MAY-01 VOC-20DEC-01 VOC-21APR-02 VUI-21FEB-03 VUI-21APR-01 E484K VUI-21JUL-01 VUI-21JAN-01 VUI-21MAR-02 VUI-21MAY-02 VUI-21JUN-01 VOC-21JAN-02 VUI-21APR-03 VOC-21FEB-02

Coverage Plots (Passed Reads)

barcode15 | Log Coverage

Select a barcode from the select box and then highlight a region on this coverage chart to see it in detail below.

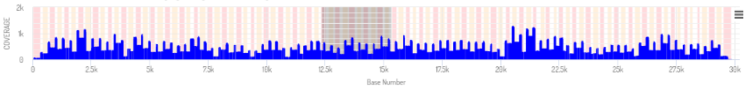

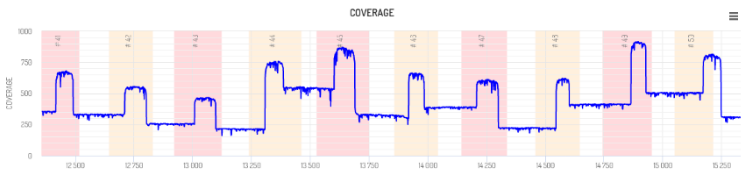

barcode87 Results Summary

Summary of barcode data.

Avg. Coverage	438
Var. Coverage	48615.31
Min. Coverage	0
Max. Coverage	1299
% reads in run	3.42
Has Finished	True
Has Sufficient Coverage	True

Download Results Files | Re-run Artic Command

Pangolin Lineage Assignments.

	0
taxon	barcode87/ARTIC/medaka_MN908947.3
lineage	B.1.1.7
conflict	0.0

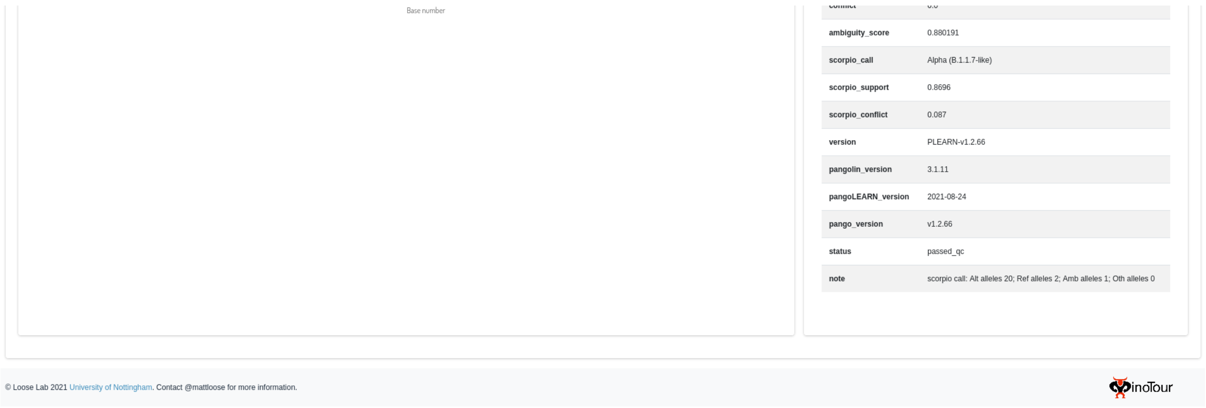

Supplementary figure 10 - ARTIC Pipeline results. This page visualises the output of the ARTIC pipeline built into minoTour, showing metrics per sample, lineage for completed samples and variants and a simple phylogenetic tree for all lineages.

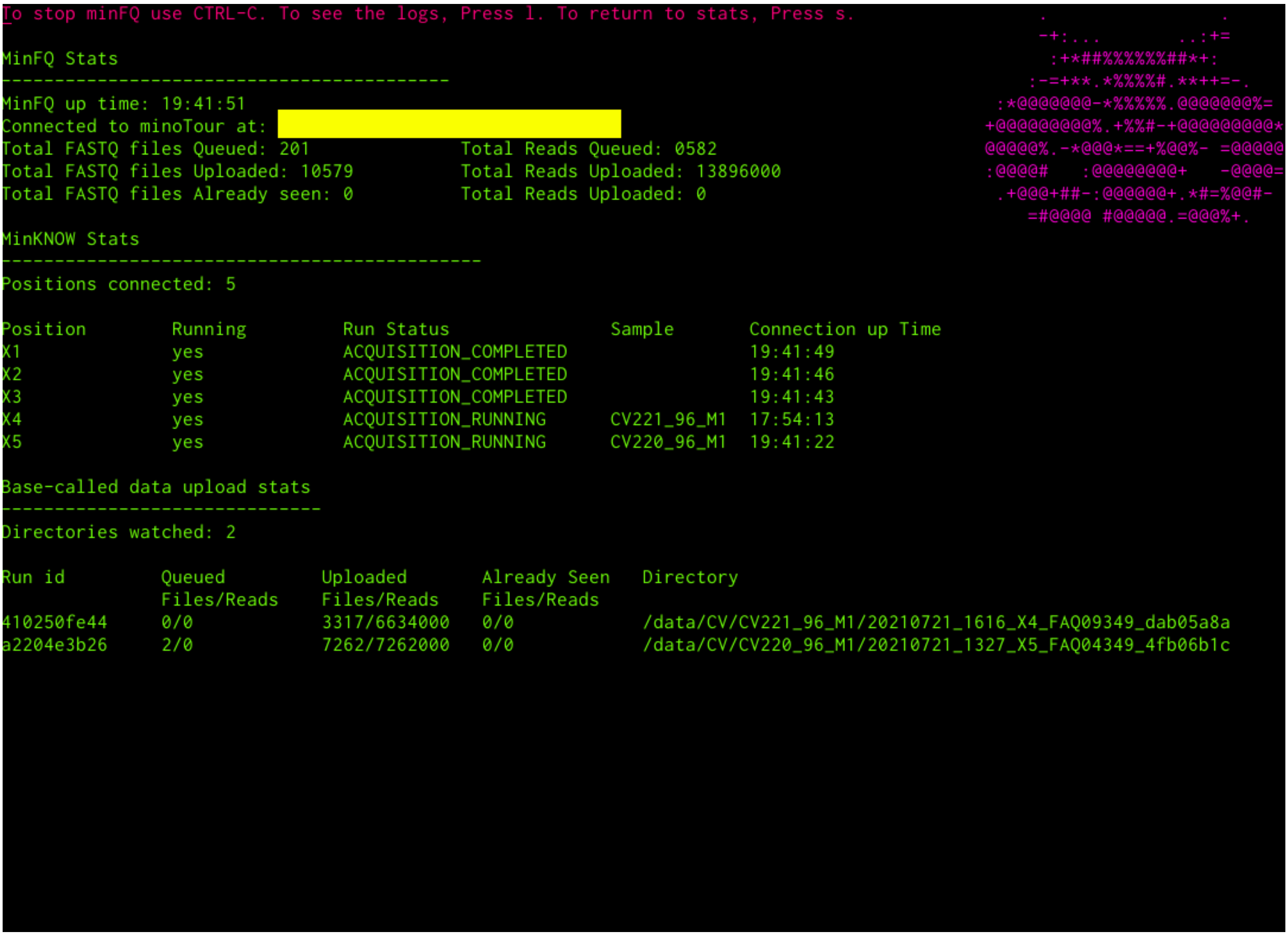

Supplementary figure 11 - MinFQ terminal interface. The minFQ interface here lists upload statistics for live monitoring and for base-called data automatically detected from the run.
